# Quadruple Multiple Primary Malignancies: Early Detection of Second Primary Malignancy by Esophagogastroduodenoscopy/Colonoscopy Is Crucial for Patients with Classic Kaposi’s Sarcoma

**DOI:** 10.3390/diagnostics10040218

**Published:** 2020-04-14

**Authors:** Nobuyuki Maruyama, Yuko Okubo, Masato Umikawa, Akiko Matsuzaki, Akira Hokama, Fusahiro Hirano, Tessho Maruyama, Kazuhide Nishihara, Toshiyuki Nakasone, Shoko Makishi, Hiroyuki Nakamura, Naoki Yoshimi

**Affiliations:** 1 Department of Oral and Maxillofacial Functional Rehabilitation, Graduate School of Medicine, University of the Ryukyus, 207 Uehara, Nishihara, Okinawa 903-0215, Japan; k178750@eve.u-ryukyu.ac.jp (N.M.); kazuhide@med.u-ryukyu.ac.jp (K.N.); makisi@med.u-ryukyu.ac.jp (S.M.); hnak@med.u-ryukyu.ac.jp (H.N.); 2 Department of Dermatology, Graduate School of Medicine, University of the Ryukyus, 207 Uehara, Nishihara, Okinawa 903-0215, Japan; yuko-okubo@live.jp; 3 Department of Medical Biochemistry, Graduate School of Medicine, University of the Ryukyus, 207 Uehara, Nishihara, Okinawa 903-0215, Japan; umikawa@med.u-ryukyu.ac.jp; 4 Department of Pathology, Ryukyu University Hospital, 207 Uehara, Nishihara, Okinawa 903-0215, Japan; ammatsu@med.u-ryukyu.ac.jp (A.M.); yoshimi@med.u-ryukyu.ac.jp (N.Y.); 5 Department of Endoscopy, Ryukyu University Hospital, 207 Uehara, Nishihara, Okinawa 903-0215, Japan; hokama-a@med.u-ryukyu.ac.jp; 6 Department of Oral and Maxillofacial Surgery, Ryukyu University Hospital, 207 Uehara, Nishihara, Okinawa 903-0215, Japan; fusahilotte3@yahoo.co.jp (F.H.); nakasone4266@gmail.com (T.N.); 7 Molecular Microbiology Group, Tropical Biosphere Research Center, University of the Ryukyus, Senbaru 1, Nishihara, Okinawa 903-0213, Japan; 8 Department of Pathology and Oncology, Graduate School of Medicine, University of the Ryukyus, 207 Uehara, Nishihara, Okinawa 903-0215, Japan

**Keywords:** kaposi’s sarcoma, second primary malignancy, visceral, upper endoscopy, esophagogastroduodenoscopy, colonoscopy

## Abstract

Currently, Kaposi’s sarcoma (KS) is treated following the recommendations of international guidelines. These guidelines recommend esophagogastroduodenoscopy/colonoscopy for detecting multicentric KS of visceral lesions. Second primary malignancies (SPMs) are also a common KS complication; however, information on their detection and treatment is unfortunately not yet indicated in these guidelines. This paper reports on an 86-year-old man who suffered from quadruple primary malignancies: skin classic KS with colon adenocarcinoma, oral squamous cell carcinoma (maxilla), and well-differentiated stomach adenocarcinoma. Gastric cancer was incidentally detected during esophagogastroduodenoscopy, which was performed to detect visceral KS. We suggest that esophagogastroduodenoscopy/colonoscopy be routinely performed during the follow-up of patients with KS. As SPMs are crucial complications in patients with KS, these malignancies should be detected as early as possible.

## 1. Introduction

Kaposi’s sarcoma (KS) was first described by Moriz Kaposi in 1872 [1]. Currently, KS is classified into four types: classic KS (CKS), endemic KS, iatrogenic KS, and epidemic KS (associated with acquired immunodeficiency syndrome) [2]. This type of tumor is very rare; however, KS is prevalent worldwide, comprising 44,000 incident cases and 27,000 deaths globally in 2012 [3]. Among the four types, CKS is relatively indolent and displays a good treatment response [4]. Lesions usually develop on the skin; however, CKS also tends to develop multifocally, such as via a visceral spread [5,6,7]. Therefore, upper endoscopy, i.e., esophagogastroduodenoscopy (EGD)/colonoscopy (CS), is suggested for detecting multicentric visceral lesions according to the latest National Comprehensive Cancer Network (NCCN) guidelines for patients with KS; however, the follow-up term is not defined [8].

For patients with KS, second primary malignancies (SPMs) are a serious problem [9,10]. SPMs in patients with KS can occur > 15 years after the CKS diagnosis [11,12]. However, the detection method of SPMs is not yet indicated in the guidelines [8]. Here, we report an 86-year-old man who suffered from quadruple primary malignancies (PMs): colon adenocarcinoma, skin CKS, oral squamous cell carcinoma (SCC) (maxilla), and well-differentiated stomach adenocarcinoma. Among these tumors, gastric cancer was incidentally detected during EGD performed to detect visceral KS. To our knowledge, this is the first known case with this combination of quadruple PMs.

## 2. Case Report

**Figure 1:**
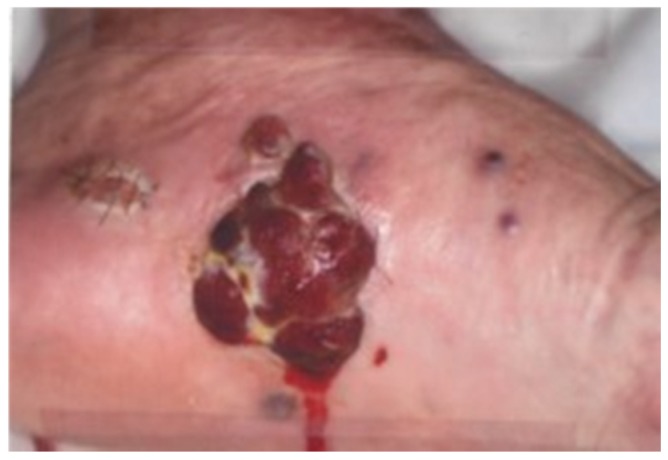
Physical examination indicated a 50 × 60 mm pedunculated red mass on the sole of the patient’s left foot.

An 86-year-old male patient first noticed a lesion 8 months prior to the visit and underwent several biopsies at the Okinawa Prefectural Miyako Hospital (Okinawa, Japan). Angiosarcoma was histologically suspected, and he was subsequently referred to the Department of Dermatology, Ryukyu University Hospital, in August 2007. A physical examination indicated a 50 × 60 mm pedunculated red mass at the sole of his left foot (Figure 1) with partial ulceration. Other dark 5 mm red papules were scattered on the sole of his left foot and left lower leg regions. The patient was surgically treated for colon adenocarcinoma (the first malignancy, September 2002) but had never undergone radiation therapy or chemotherapy. The patient was a previous smoker (2–3 packs per day for 10 years) in his 20s and 30s with a history of drinking. No incidence of cancer was observed in his family history. The patient also suffered from cerebral infarction and Ménière’s disease. Subsequently, the patient underwent biopsy of the foot lesion, and angiosarcoma was histologically suspected. A DNA-polymerase chain reaction test of the frozen tissue was also performed, which tested positive for human herpesvirus 8. He was a human T-cell leukemia virus type 1 carrier. The serological test was negative for human immunodeficiency virus-1 and virus-2. Therefore, he was clinically and pathologically diagnosed with CKS. Tumor excision of the sole of his left foot was performed under spinal anesthesia (September 2007). Postoperatively, EGD and CS were performed to detect the presence of multicentric CKS and to confirm a postoperative colon lesion. Considering his old age, postoperative radiation was avoided, and an imiquimod cream treatment was continued at Miyako Hospital after discharge. The follow-up EGD was done in December 2007, and no tumorous lesion was found.

The third malignancy, i.e., oral SCC, developed 3 years after the CKS surgery. The patient was referred to the Department of Oral and Maxillofacial Surgery at the Ryukyu University Hospital in September 2010 for further evaluation of the right gum of his upper jaw, in which a 22 × 18 mm mass was located. An EGD demonstrated no tumorous data. A well-differentiated SCC in the maxilla was pathologically diagnosed based on the oral mass biopsy. Neoadjuvant chemotherapy and surgical resection of the oral cancer were planned. The neoadjuvant chemotherapy included bleomycin (75 mg total) and “uracil and tegafur” (450 mg per day for approximately 1 month) and displayed a partial response in the tumor; however, the patient refused to undergo subsequent resection of the oral cancer and refused continuous chemotherapy. The patient was discharged and scheduled for follow-up for his oral cancer at Okinawa Prefectural Miyako Hospital.

**Figure 2:**
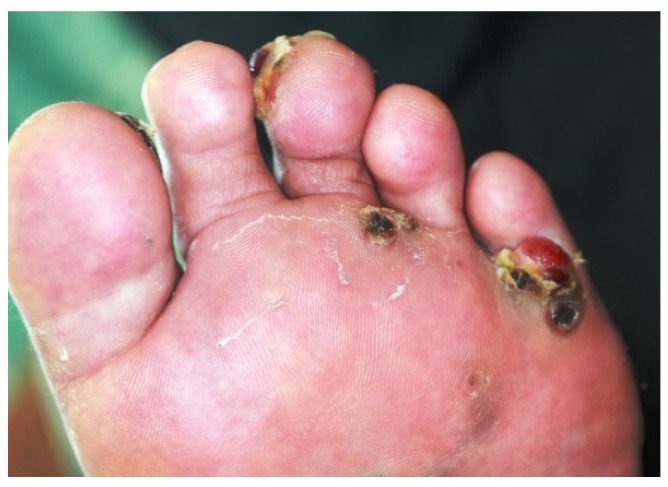
Physically, tumor nodules had expanded and ulcerated on both soles of his feet and his right thigh. (Image of the left sole).

**Figure 3:**
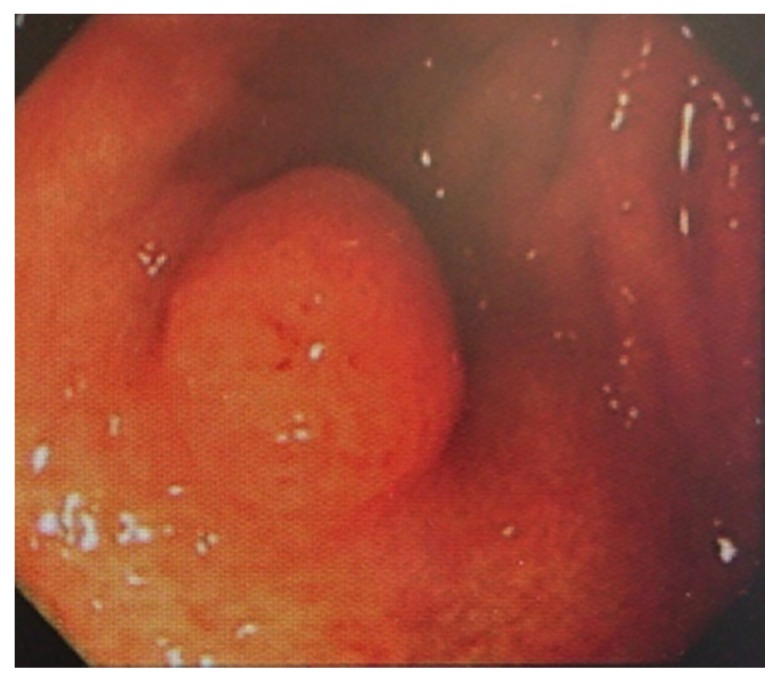
A 1 cm mass was newly found in the patient’s stomach through esophagogastroduodenoscopy. Biopsy of the mass histologically revealed a high-grade (group 4) gastric adenoma, which was also suspected as gastric cancer.

Although the patient continued to receive treatment for his KS with imiquimod cream at the Okinawa Prefectural Miyako Hospital, his KS exacerbated. Therefore, he was referred again to our institute in June 2011 for further evaluation and treatment. Physically, his tumor nodules had expanded and ulcerated on both soles of his feet and his right thigh (Figure 2). To detect metastasis or multicentric KS, several tests were performed. In regard to the oral cancer (the third malignancy), it was progressing slowly, without palpable neck lymph nodes. Computed tomography (CT) scan showed no lung tumorous lesion; however, interstitial pneumonia was radiologically detected, with suspected bleomycin-induced interstitial pneumonia. Considering his age (>90 years) and the lung lesion, the patient could not undergo chemotherapy for CKS. Therefore, the CKS was treated with 5% imiquimod cream, cryotherapy, and tumor-debulking surgery using an electric knife (June 2011). No exacerbated lesions were identified after the CKS treatment. According to the EGD finding, no multicentric CKS lesions were identified; however, a 1 cm mass was newly observed in his stomach by the EGD. A mass biopsy histologically revealed a high-grade (group 4) gastric adenoma, which was also suspected as gastric cancer (Figure 3). Serum tests for anti-*Helicobacter pylori* antibodies were negative. Therefore, endoscopic submucosal dissection was performed (July 2011), diagnosing a well-differentiated adenocarcinoma Type IIa (the fourth malignancy) histologically. No venous or lymphatic invasion was histologically found. The follow-up EGD was done in September 2011, and no tumorous lesion was found. Oral cancer examination revealed progression of the oral mass and mandibular lymphadenopathy. However, no treatment was administered due to the patient’s advanced age. No evidence of lung metastasis was found. In July 2012, oral cancer continued to progress, his condition continued to worsen, and he died of oral cancer at the Okinawa Prefectural Miyako Hospital. This report was submitted for ethical review to the Ethics Committee of the University of the Ryukyus (Okinawa, Japan), which waived the requirement for review per institutional protocol because the study did not contain content that requires ethical approval. The Ethics Committee approved the submission and publication of the manuscript in April 27, 2018. Written informed consent was obtained from the patient’s kin for the publication of this case report and the accompanying images. A copy of the written consent is available for review from the Editor-in-Chief of this journal.

## 3. Discussion

The following two important issues were noted in this case: (i) to our knowledge, the combination of quadruple PMs (colon adenocarcinoma, skin CKS, well-differentiated SCC of the oral cavity, and gastric well-differentiated adenocarcinoma) has not been reported, and (ii) we suggest that EGD/CS be routinely performed to detect SPMs during the long-term follow-up of patients with KS.

The current case was diagnosed as quadruple PMs using Warren and Gates criteria [13]. To identify tumor combinations similar to our case, the literature from 1872 [1] to 2020 was searched using PubMed and Google Scholar. Non-English studies and English conference proceedings were excluded. A total of 280 cases of patients with KS with multiple tumors including head and neck, esophagus, stomach, duodenum, or colorectal (including anal) malignancies were identified [7,10,11,12,14,15,16,17,18,19,20,21,22,23,24,25,26,27,28,29] (Table 1). Of the identified cases, 7, 20, 1, and 159 had KS with esophagus, stomach, duodenum, and colorectal (including anal) malignancy, respectively. However, no previous cases were similar to the current combination of tumors. We then attempted to investigate how these tumors were detected (by clinical symptoms, radiological methods, or EGD/CS) in the literature presented in Table 1; however, detection methods could not be found because most of the identified studies were based on cancer registries that did not describe the detailed case information [10,11,17,28].

We believe that life-threatening SPMs should be detected in the early stage in patients with CKS. In our case, early-stage gastric cancer was accidentally detected by EGD (3 years and 9 months after the treatment of CKS). However, no universal guidelines (NCCN) exist for patients with CKS. Conversely, European consensus-based interdisciplinary guidelines state that endoscopy is not strongly recommended for patients with CKS [30]. In these guidelines, endoscopy (as well as bronchoscopy and total-body CT) is recommended to be performed just according to symptoms in both staging work-up and follow-up for patients with all types of KS [30]. This raises the question as to why endoscopy does not appear to be important in the examination of patients with CKS. The following two reasons have been indicated: (i) According to some studies, visceral involvement of CKS was reported to be only <10%, which is apparently less than that of other KS [7,30]. Further, a recent report stated that the follow-up interval for patients with CKS can be every 6–12 months, essentially based on clinical examination [31]. (ii) SPMs for patients with CKS are not fully considered. In fact, Cesarman et al. mentions that the early detection of KS and other cancers is beneficial for patients with KS, but these benefits have not been examined in randomized trials [31]. As the recent reports state, the goal of CKS therapy is to manage disease control and preserve quality of life [30,31], and to achieve this goal, life-threatening SPMs should be detected in early stages in patients with CKS.

Despite no clinical gastric/colorectal symptoms, EGD/CS was performed in the current case for two reasons: first, to detect multicentric CKS of the visceral regions, and second, to examine colon cancer recurrence because the patient had a history of colon adenocarcinoma. Consequently, gastric SPM was incidentally detected. For patients with KS, SPMs as well as multicentric KS in other sites are reportedly serious complications [10,28]. Based on our literature review, the first case of SPM (lymphatic leukemia) in a patient with KS was reported in 1920 [32]. Subsequently, SPMs associated with KS were debated in 1954 [9]. Then, in 1973, it was reported that SPM (stomach cancer) as well as visceral involvement could be the cause of death in a KS patient [16]. Lymphoreticular malignancies were reported to tend to occur as SPMs in 1980 [33]. After that, other reports described “solid tumors” that also occurred in patients with KS as SPMs [6,34]. KS is a rare disease; therefore, patients in previous studies should be reviewed to identify its characteristics [35]. Table 1 shows that more than 280 patients with KS (including our case) had solid malignancy associated with head and neck, esophagus, stomach, duodenum, or colorectal cancer. KS itself has a relatively good prognosis [36]. In fact, according to the mortality rate of patients with KS, SPMs were higher than in those with KS alone as a cause of death [7,14,18,20,37]. However, the newest NCCN guidelines on KS (HIV-related) only describe that radiation is a risk of SPMs; they do not describe methods to detect such tumors [8], potentially because most HIV-related patients with KS die from HIV itself [28]. Conversely, CKS (non-HIV-related KS) is a slow, indolent disease that has a relatively good prognosis [2,4]. Indeed, EGD (which revealed gastric cancer) did not appear to affect the survival of the current patient due to the early stage of the tumor. Moreover, the patient exhibited no visceral KS. Based on a literature review, we suggest routine EGD because both visceral lesions and SPM can occur in patients with CKS. Weissmann et al. reported that visceral KS and SPM occurred in 18.4% and 16.8%, respectively, of 125 studied patients with CKS [20]. Among 56 patients with CKS studied by Errihani et al., visceral KS and SPM occurred in 16.1% and 10.7%, respectively [24]. Among the cases of visceral KS, 22% occurred in the gastrointestinal tract [24]. Although these reported rates are not high, visceral CKS, SPM, or both can occur in patients with CKS. According to the descriptions provided in Table 1, SPM can occur in the esophagus, stomach, and duodenum. Therefore, as described above, to prevent death from SPMs, they should be detected in patients with CKS, such as our case.

We think that if clinicians use the guidelines for patients with KS, SPMs cannot be detected at an early stage because clinical symptoms are basically expressed in advanced-stage disease (poor outcome) [38,39]. Table 1 shows that gastric or colorectal SPM can occur in patients with KS. These cancers should be detected as early as possible because early-stage cancer has a good prognosis; by contrast, advanced cases have a poor prognosis [40,41]. However, as described above, the newest NCCN guidelines for patients with KS do not recommend regular follow-up with EGD/CS [8]. The guidelines report that EGD/CS is useful when patients with KS have gastrointestinal symptoms or positive hemoccult with suspected visceral KS involvement (i.e., multicentric KS of the other site) [8]. However, the guidelines do not indicate detecting SPMs in patients with KS. Furthermore, SPMs in patients with CKS can occur metachronously from 1 to >15 years after the CKS diagnosis [11,12]. Iscovich et al. reported that in 37.7% (*n* = 23) of 61 patients, SPM occurred ≥5 years after the diagnosis of CKS [12]. Hiatt et al. reported that 42% (*n* = 45) of 108 patients with CKS had SPM on long-term follow-up (<1 to 19 years; average = 4.8 years) [7]. Remarkably, 24% of patients died of SPM, whereas only 4% died of CKS [7]. Hjalgrim et al. reported that as SPM of CKS, 35 involved the digestive organs and peritoneum (*n* = 35); among these, 82.9% (*n* = 29) occurred ≥ 1 year after the diagnosis of CKS [11]. Among the 35 patients, SPMs in the colon were found in 14 patients. Among them, 92.9% (*n* = 13) experienced SPM > 1 year after the diagnosis of CKS [11]. No cancerous lesion was found in the current case on EGD 3 years after primary KS treatment; however, the next EGD (3 years and 9 months after treatment of the primary KS) detected gastric cancer. Based on the current case and a literature review, we suggest that follow-up EGD/CS should be conducted at least 1 year after the diagnosis of CKS, and follow-up EGD/CS after 3 years may be reasonable. Therefore, long-term follow-up with EGD/CS is necessary for patients with CKS after the treatment.

To detect SPMs or visceral KS in patients with KS, EGD/CS is a more useful approach than positron-emission tomography (PET)/CT. PET/CT has been well-reported to detect SPMs in various body sites of patients with primary cancer [42,43,44,45]. However, we recommend EGD/CS for these patients for the following reasons: First, PET/CT may overlook SPMs because it potentially overlooks small-sized upper gastrointestinal cancer and colon cancer. Yabuki et al. [46] and Suzuki et al. [47] reported that PET or PET/CT tended to overlook the stages (T2, T1, and Tis) of upper gastrointestinal cancer because of the low accumulation. By contrast, esophagogastroscopy could detect cases of cancer that were overlooked by PET/CT [46]. Furthermore, small-sized colorectal lesions were significantly missed by PET/CT based on 492 patients who underwent both PET/CT and CS [48]. Further, the physiological uptake on the intestine masks colorectal lesions [49]. Conversely, EGD/CS has been reported to be useful for patients with KS. Kim et al. reported the low detection ability of PET/CT for esophageal and stomach SPMs after the treatment of head and neck SCC [50]. By contrast, for patients with KS, EGD could detect hidden gastrointestinal lesions that were overlooked by the preceding PET/CT [51]. Second, small-sized visceral KS may be overlooked by PET/CT in patients with KS [52]. Third, SPMs should be detected as early as possible. Certainly, EGD/CS is more invasive and stressful than PET/CT [53,54,55]. However, as in the current case, skin and oral lesions could be directly observed, whereas stomach and colon lesions should be detected and observed using an appropriate method.

## 4. Conclusions

We report a rare case of CKS associated with quadruple PMs. We suggest that EGD/CS should be routinely performed during the follow-up of patients with KS. As SPMs are a crucial complication in patients with KS, they should be detected as early as possible.

## Figures and Tables

**Table 1:** Cases of combined Kaposi’s sarcoma (KS) and second primary malignancies (SPMs) of the head and neck, esophagus, stomach, duodenum, or colorectum (including anal).

Ref.	Year	Type of KS	Type of SPM	Number of Cases	Patient Background (Institutes or Register Data)
[14]	1966	KS	Tongue carcinoma	1	Memorial Hospital for Cancer and Allied Diseases, USA
[14]	1966	KS	Tonsil carcinoma	1	
[14]	1966	KS	Colon carcinoma	1	
[15]	1967	KS	Mouth SCC	1	Mayo Clinic, USA
[16]	1973	KS	Stomach cancer	2	Tel Aviv University Medical School, Beilinson Medical Center, Petah Tikva
[16]	1973	KS	Rectal cancer	1	
[17]	1996	KS	Stomach	1	1 of 11 population-based Italian Cancer Registries, Italy
[18]	1997	CKS	Larynx carcinoma	1	National University of Athens, Greece
[11]	1997	CKS	Buccal cavity and pharynx cancer	5	The Nordic Cancer Registries (Norway, Denmark, Sweden, Finland, and Iceland)
[11]	1997	CKS	Colon cancer	14	
[19]	1999	CKS	Oral cavity	5	The Israel Cancer Registry (SPMs before KS), Israel (1961–1992)
[19]	1999	CKS	Stomach	1	
[19]	1999	CKS	Colon, sigma	5	
[19]	1999	CKS	Ampulla, anus	6	
[19]	1999	CKS	Central nervous system	2	
[19]	1999	CKS	Thyroid	1	
[12]	1999	CKS	Stomach	3	The Israel Cancer Registry (SPMs after KS), Israel (1961–1992)
[12]	1999	CKS	Colon	6	
[12]	1999	CKS	Rectum	9	
[12]	1999	CKS	Esophagus	1	
[12]	1999	CKS	Larynx	2	
[12]	1999	CKS	Brain and central nervous system	2	
[20]	2000	CKS	Adenoma of colon ^c^	3	Rambam Medical Center in Haifa, Israel (1957–1993) ^a^
[20]	2000	CKS	SCC of vocal cords	1	
[20]	2000	CKS	Adenoma of buccal mucosa ^c^	1	
[21]	2005	CKS	Mouth angiosarcoma	1	Universita` Cattolica del Sacro Cuore, Italy
[22]	2005	CKS	Parotid oncocytoma	1	Universidad Peruana Cayetano Heredia, Peru
[22]	2005	CKS	Colon adenocarcinoma	1	
[22]	2005	CKS	Cancer of the appendix	1	
[23]	2006	KS	Nasal mucosa cancer	1	The North Italian Transplant Program, Italy
[7]	2008	CKS	Colorectal carcinoma	3	The Soft Tissue Pathology Registry at the Armed Forces Institute of Pathology, USA
[7]	2008	CKS	Laryngeal carcinoma	2	
[7]	2008	CKS	Oral SCC	1	
[7]	2008	CKS	Gastric carcinoma	1	
[24]	2011	CKS	Colic cancer	1	National Institute of Oncology, Rabat, Morocco
[25]	2014	AIDS-associated KS	Duodenal adenocarcinoma; prostatic adenocarcinoma	1	The Doubs Cancer Registry belongs to the FRANCIM association (French Network of Cancer Registries)
[25]	2014	CKS	Gastric carcinoid tumor	1	
[25]	2014	CKS	Cerebral glioblastoma	1	
[25]	2014	Iatrogenic KS	Follicular lymphoma of the parotid	1	
[26]	2014	AIDS-associated KS	Penis; lip; oral cavity	1	Instituto Nacional de Cancerologı’a, Mexico
[27]	2015	KS	Gastric neoplasm of mucosa-associated lymphoid tissue	1	University of Naples Federico II, Italy
[28]	2017	KS	Oral cavity and pharynx cancer	23	SEER, USA (1981–2013) ^b^
[28]	2017	KS	Tongue cancer	11	
[28]	2017	KS	Esophageal cancer	6	
[28]	2017	KS	Stomach cancer	10	
[28]	2017	KS	Colon and rectum cancer	45	
[28]	2017	KS	Anal carcinoma	62	
[28]	2017	KS	Larynx cancer	4	
[28]	2017	KS	Brain cancer	6	
[10]	2018	KS	Ascending colon	4	SEER, USA (data from only 1973–1979 were used) ^b^
[10]	2018	KS	Colon	13	
[29]	2019	KS	Invasive laryngeal SCC; B-cell non-Hodgkin’s lymphoma in tongue root and lymph node	1	Department of Otorhinolaryngology, Bagcilar Training and Research Hospital, Istanbul, Turkey
The current report	2020	CKS (current case)	Colon adenocarcinoma; oral SCC (maxilla); well-differentiated stomach adenocarcinoma	1	Ryukyu University Hospital, Nishihara, Okinawa, Japan

AIDS, acquired immunodeficiency syndrome; CKS, classic Kaposi’s sarcoma; SCC, squamous cell carcinoma; SEER, surveillance, epidemiology, and end results; SPM, second primary malignancy; KS, Kaposi’s sarcoma. ^a^ It is unknown why cases from ref. 20 overlapped with ref. 19 or 12. ^b^ Both ref. 28 (1981–2013) and ref. 10 (1973–2013) used SEER research data. We chose most cases from ref. 28 due to the availability of more detailed data. Data from 1973 to 1979 could be used; however, data from 1980 could not be used. ^c^ The tumor is reported as malignancy (according to ref. 20).

## References

[1] Kaposi M. (1872). Idiopathisches multiples pigmentsarkom der haut. Arch. Dermatol. Syph..

[2] Patrikidou A., Vahtsevanos K., Charalambidou M., Valeri R.M., Xirou P., Antoniades K. (2009). Non-AIDS Kaposi’s sarcoma in the head and neck area. Head Neck.

[3] Ferlay J., Soerjomataram I., Dikshit R., Eser S., Mathers C., Rebelo M., Rebelo M., Parkin D.M., Forman D., Bray F. (2015). Cancer incidence and mortality worldwide: Sources, methods and major patterns in GLOBOCAN 2012. Int. J. Cancer.

[4] Piette W.W. (1987). The incidence of second malignancies in subsets of Kaposi’s sarcoma. J. Am. Acad. Dermatol..

[5] Hengge U.R., Ruzicka T., Tyring S.K., Stuschke M., Roggendorf M., Schwartz R.A., Seeber S. (2002). Update on Kaposi’s sarcoma and other HHV8 associated diseases. Part 1: Epidemiology, environmental predispositions, clinical manifestations, and therapy. Lancet. Infect. Dis..

[6] Brenner B., Weissmann-Brenner A., Rakowsky E., Weltfriend S., Fenig E., Friedman-Birnbaum R., Sulkes A., Linn S. (2002). Classical Kaposi sarcoma: Prognostic factor analysis of 248 patients. Cancer.

[7] Hiatt K.M., Nelson A.M., Lichy J.H., Fanburg-Smith J.C. (2008). Classic Kaposi Sarcoma in the United States over the last two decades: A clinicopathologic and molecular study of 438 non-HIV-related Kaposi Sarcoma patients with comparison to HIV-related Kaposi Sarcoma. Mod. Pathol..

[8] (2020). National Comprehensive Cancer Network: AIDS-Related Kaposi Sarcoma Version 1. https://www.nccn.org/professionals/physician_gls/pdf/kaposi.pdf.

[9] Alien A.C. (1954). Chapter XXVII tumors of vessels. The Skin. A Clinicopathologic Treatise.

[10] Mukhtar F., Ilozumba M., Utuama O., Cimenler O. (2018). Change in pattern of secondary cancers after kaposi sarcoma in the era of antiretroviral therapy. JAMA Oncol..

[11] Hjalgrim H., Frisch M., Pukkala E., Tulinius H., Ekbom A., Dictor M., Langmark F., Hardarson S., Melbye M. (1997). Risk of second cancers in classical Kaposi’s sarcoma. Int. J. Cancer.

[12] Iscovich J., Boffetta P., Brennan P. (1999). Classic Kaposi’s sarcoma as a first primary neoplasm. Int. J. Cancer.

[13] Warren S., Gates O. (1932). Multiple primary malignant tumors: A survey of the literature and a statistical study. Am. J. Cancer.

[14] O’Brien P.H., Brasfield R.D. (1966). Kaposi’s sarcoma. Cancer.

[15] Moertel C.G., Dockerty M.B., Baggenstoss A.H. (1961). Multiple primary malignant neoplasms. I. Introduction and presentation of data. Cancer.

[16] Feuerman E.J., Potruch-Eisenkraft S. (1973). Kaposi’s sarcoma. A follow-up of 38patients. Dermatologica.

[17] Franceschi S., Arniani S., Balzi D., Geddes M. (1996). Survival of classic Kaposi’s sarcoma and risk of second cancer. Br. J. Cancer.

[18] Stratigos J.D., Potouridou I., Katoulis A.C., Hatziolou E., Christofidou E., Stratigos A., Hatzakis A., Stavrianeas N.G. (1997). Classic Kaposi’s sarcoma in Greece: A clinico-epidemiological profile. Int. J. Dermatol..

[19] Iscovich J., Boffetta P., Winkelmann R., Brennan P. (1999). Classic Kaposi’s sarcoma as a second primary neoplasm. Int. J. Cancer.

[20] Weissmann A., Linn S., Weltfriend S., Friedman-Birnbaum R. (2000). Epidemiological study of classic Kaposi’s sarcoma: A retrospective review of 125 cases from Northern Israel. J. Eur. Acad. Dermatol. Venereol..

[21] Gambassi G., Semeraro R., Suma V., Sebastio A., Incalzi R.A. (2005). Aggressive behavior of classical Kaposi’s sarcoma and coexistence with angiosarcoma. J. Gerontol. A Biol. Sci. Med. Sci..

[22] Mohanna S., Ferrufino J.C., Sanchez J., Bravo F., Gotuzzo E. (2005). Epidemiological and clinical characteristics of classic Kaposi’s sarcoma in Peru. J. Am. Acad. Dermatol..

[23] Taioli E., Piselli P., Arbustini E., Boschiero L., Burra P., Busnach G., Caldara R., Citterio F., De Juli E., Dissegna D. (2006). Incidence of second primary cancer in transplanted patients. Transplantation.

[24] Errihani H., Berrada N., Raissouni S., Rais F., Mrabti H., Rais G. (2011). Classic Kaposi’s sarcoma in Morocco: Clinico-epidemiological study at the National Institute of Oncology. BMC Dermatol..

[25] Laresche C., Fournier E., Dupond A.S., Woronoff A.S., Drobacheff-Thiebaut C., Humbert P., Aubin F. (2014). Kaposi’s sarcoma: A population-based cancer registry descriptive study of 57 consecutive cases diagnosed between 1977 and 2009. Int. J. Dermatol..

[26] Volkow P., Lizano M., Carrillo-García A., Pérez-Montiel D., Garciadiego P. (2014). Triple secondary neoplasms: Penis, lip and oral cavity in an AIDS patient treated with pegylated liposomal doxorubicin for cutaneous Kaposi’s sarcoma. AIDS.

[27] Santangelo M.L., Criscitiello C., Renda A., Federico S., Curigliano G., Dodaro C., Scotti A., Tammaro V., Calogero A., Riccio E. (2015). Immunosuppression and Multiple Primary Malignancies in Kidney-Transplanted Patients: A Single-Institute Study. Biomed. Res. Int..

[28] Kumar V., Garg M., Chaudhary N., Soni P., Floudas C.S., Nwanyanwu C., Chandra A. (2017). The pattern of secondary cancers in patients with Kaposi sarcoma in the United States. Cancer Causes Control.

[29] Yildirim M., Belli S., Ozsoy S., Taskin U. (2019). Primary triple head and neck tumors: Laryngeal squamous cell carcinomas, Kaposi’s sarcoma, and non-Hodgkin’s lymphoma. Indian. J. Pathol. Microbiol..

[30] Lebbe C., Garbe C., Stratigos A.J., Harwood C., Peris K., Marmol V.D., Malvehy J., Zalaudek I., Hoeller C., Dummer R. (2019). Diagnosis and treatment of Kaposi’s sarcoma: European consensus-based interdisciplinary guideline (EDF/EADO/EORTC). Eur. J. Cancer.

[31] Cesarman E., Damania B., Krown S.E., Martin J., Bower M., Whitby D. (2019). Kaposi sarcoma. Nat. Rev. Dis. Primers..

[32] Cole H.N., Crump E.S. (1920). Report of two cases of idiopathic hemorrhagic sarcoma (Kaposi), the first complicated with lymphatic leukemia. Arch. Dermatol. Syph..

[33] Safai B., Miké V., Giraldo G., Beth E., Good R.A. (1980). Association of Kaposi’s sarcoma with second primary malignancies: Possible etiopathogenic implications. Cancer.

[34] Iscovich J., Boffetta P., Franceschi S., Azizi E., Sarid R. (2000). Classic Kaposi sarcoma: Epidemiology and risk factors. Cancer.

[35] Awazawa R., Utsumi D., Katano H., Awazawa T., Miyagi T., Hayashi K., Matori S., Uezato H., Takahashi K. (2017). High prevalence of distinct human herpesvirus 8 contributes to the high incidence of non-acquired immune deficiency syndrome-associated kaposi’s sarcoma in isolated Japanese islands. J. Infect. Dis..

[36] Kaposi’s Sarcoma-Cancer Statistics Review 1975–2015. https://seer.cancer.gov/csr/1975_2015/results_merged/sect_10_kaposi_sarcoma.pdf.

[37] Donin N., Filson C., Drakaki A., Tan H.J., Castillo A., Kwan L., Litwin M., Chamie K. (2016). Risk of second primary malignancies among cancer survivors in the United States, 1992 through 2008. Cancer.

[38] Moslim M.A., Heald B., Tu C., Burke C.A., Walsh R.M. (2018). Early genetic counseling and detection of CDH1 mutation in asymptomatic carriers improves survival in hereditary diffuse gastric cancer. Surgery.

[39] Hatch Q.M., Kniery K.R., Johnson E.K., Flores S.A., Moeil D.L., Thompson J.J., Maykel J.A., Steele S.R. (2016). Screening or symptoms? How do we detect colorectal cancer in an equal access health care system?. J. Gastrointest. Surg..

[40] O’Connell J.B., Maggard M.A., Ko C.Y. (2004). Colon cancer survival rates with the new American Joint committee on cancer sixth edition staging. J. Natl. Cancer Inst..

[41] Sano T., Coit D.G., Kim H.H., Roviello F., Kassab P., Wittekind C., Yamamoto Y., Ohashi Y. (2017). Proposal of a new stage grouping of gastric cancer for TNM classification: International gastric cancer association staging project. Gastric. Cancer.

[42] Kim Y., Roh J.L., Kim J.S., Lee J.H., Choi S.H., Nam S.Y., Kim S.Y. (2019). Chest radiography or chest CT plus head and neck CT versus (18)F-FDG PET/CT for detection of distant metastasis and synchronous cancer in patients with head and neck cancer. Oral. Oncol..

[43] Moletta L., Bissoli S., Fantin A., Passuello N., Valmasoni M., Sperti C. (2018). PET/CT incidental detection of second tumor in patients investigated for pancreatic neoplasms. BMC Cancer.

[44] Bang J.I., Lee E.S., Kim T.S., Kim S.K. (2015). Unexpected second primary malignancies detected by F-18 FDG PET/CT during follow-up for primary malignancy: Two case reports. Nucl. Med. Mol. Imaging.

[45] Ishimori T., Patel P.V., Wahl R.L. (2005). Detection of unexpected additional primary malignancies with PET/CT. J. Nucl. Med..

[46] Yabuki K., Kubota A., Horiuchi C., Taguchi T., Nishimura G., Inamori M. (2013). Limitations of PET and PET/CT in detecting upper gastrointestinal synchronous cancer in patients with head and neck carcinoma. Eur. Arch. Otorhinolaryngol..

[47] Suzuki H., Hasegawa Y., Terada A., Ogawa T., Hyodo I., Suzuki M., Nakashima T., Tamaki T., Nishio M. (2008). Limitations of FDG-PET and FDG-PET with computed tomography for detecting synchronous cancer in pharyngeal cancer. Arch. Otolaryngol. Head. Neck. Surg..

[48] Hirakawa T., Kato J., Okumura Y., Hori K., Takahashi S., Suzuki H., Akita M., Higashi R., Saito S., Kaji E. (2012). Detectability of colorectal neoplasia with fluorine-18-2-fluoro-2-deoxy-D-glucose positron emission tomography and computed tomography (FDG-PET/CT). J. Gastroenterol..

[49] Yasuda S., Ide M. (2005). PET and cancer screening. Ann. Nucl. Med..

[50] Kim J.W., Roh J.L., Kim J.S., Lee J.H., Cho K.J., Choi S.H., Nam S.Y., Kim S.Y. (2013). (18)F-FDG PET/CT surveillance at 3-6 and 12 months for detection of recurrence and second primary cancer in patients with head and neck squamous cell carcinoma. Br. J. Cancer.

[51] Morooka M., Ito K., Kubota K., Minamimoto R., Shida Y., Hasuo K., Ito T., Tasato D., Honda H., Teruya K. (2010). Whole-body 18F-fluorodeoxyglucose positron emission tomography/computed tomography images before and after chemotherapy for Kaposi sarcoma and highly active antiretrovirus therapy. Jpn. J. Radiol..

[52] van de Luijtgaarden A., van der Ven A., Leenders W., Kaal S., Flucke U., Oyen W., van der Graaf W. (2010). Imaging of HIV-associated Kaposi sarcoma; F-18-FDG-PET/CT and In-111-bevacizumabscintigraphy. J. Acquir. Immune Defic. Syndr..

[53] Ahn H., Shin B.S., Liang R., Pan Z., Cheok A., Haller M., Lau R.W.H., Saito H. (2006). Height-based deformation and ray supersampling for colon unfolding. Advances in Artificial Reality and Tele-Existence.

[54] Yu J.W., Park J., Song P.S., Park J.H., Kim M.S., Jeon G.J., Kim M.S., Kim T.O. (2016). Two cases of stress cardiomyopathy during esophagogastroduodenoscopy. Clin. Endosc..

[55] Perlman S.B., Hall B.S., Reichelderfer M. (2013). PET/CT imaging of inflammatory bowel disease. Semin. Nucl. Med..

